# The State of Health in Jordan, 1990–2023: A Systematic Analysis for the Global Burden of Disease Study

**DOI:** 10.1002/hsr2.73461

**Published:** 2026-10-31

**Authors:** Yazan A. Al‐Ajlouni, Jude Alawa, Abdulrahman Al‐Shamari, Aditi Hazra, Kaveh Khoshnood, Omar Shamieh, Nour Makarem, Omar Al Ta'ani, Sara Al Zubi, Adam Coutts, Basile Njei

**Affiliations:** ^1^ Department of Physical Medicine and Rehabilitation Medicine Montefiore Medical Center/Albert Einstein College of Medicine Bronx New York USA; ^2^ Yale International Medicine Program Yale University New Haven Connecticut USA; ^3^ Department of Orthopedics Hospital of Special Surgery (HSS) New York New York USA; ^4^ Weil‐Cornel School of Medicine Cornell University Doha Qatar; ^5^ Department of Medicine, Brigham and Women's Hospital Harvard Medical School Boston Massachusetts USA; ^6^ Yale School of Public Health Yale University New Haven Connecticut USA; ^7^ Department of Palliative Medicine King Hussein Cancer Center Amman Jordan; ^8^ Faculty of Medicine The University of Jordan Amman Jordan; ^9^ Department of Epidemiology, Mailman School of Public Health Columbia University, Irving Medical Center New York New York USA; ^10^ Alleghany Health Network Pittsburgh Pennsylvania USA; ^11^ Department of Medicine, Mass General Hospital Harvard Medical School Boston Massachusetts USA; ^12^ University of Cambridge Cambridge UK; ^13^ Engelhardt School of Global Health and Bioethics Euclid University Bangui Central African Republic; ^14^ Section of Digestive Diseases, Department of Medicine Yale University New Haven Connecticut USA; ^15^ VA Connecticut Healthcare West Haven CT USA; ^16^ Ohio University Heritage College of Osteopathic Medicine Athens Ohio USA; ^17^ Yale Liver Center, Yale New Haven Health New Haven Connecticut USA

**Keywords:** DALYs, disease burden, global burden of disease, Jordan, non‐communicable diseases, refugees

## Abstract

**Background:**

Jordan's health system has evolved amid rapid urbanization and the strain of hosting one of the world's largest refugee populations per capita. This study comprehensively evaluates national disease burden trends in Jordan from 1990 to 2023 to inform health priorities.

**Methods:**

Using the Global Burden of Disease (GBD) 2023 framework, we analyzed age‐standardized rates of disability‐adjusted life years (DALYs), years lived with disability (YLDs), and years of life lost (YLLs) across 370 diseases and 88 risk factors. Data were derived from national censuses, health surveys, disease registries, and hospital records, and harmonized using standardized GBD modeling and estimation procedures. Analyses were stratified by age, sex, and cause.

**Results:**

Between 1990 and 2023, age‐standardized DALYs declined by 33.88% (95% UI: −37.38 to −30.59), with greater reductions in males (−38.83%; −42.22 to −35.60) than females (−27.22%; −31.33 to −23.58). YLLs decreased by 49.38% (−50.93 to −47.68), while YLDs remained comparatively stable (2.49%; −2.37 to 7.47). Using mutually exclusive GBD Level 3 causes, ischemic heart disease and diabetes mellitus were the two leading causes of DALYs in 2023. Diabetes mellitus rose from 7th place in 1990 to 2nd place in 2023, while neonatal disorders declined from 1st to 7th place. Metabolic risks remained important contributors to the overall disease burden. Jordan's age‐standardized diabetes DALY rate in 2023 was approximately 1.56 times the global rate.

**Conclusion:**

Jordan's health profile has shifted toward a greater burden from noncommunicable diseases, while age‐standardized health loss and premature mortality have declined substantially. In contrast, nonfatal disability changed comparatively little. These findings provide an evidence base for primary prevention of NCDs and for long‐term strategies addressing the country's evolving health challenges.

## 1 Introduction

Over the past several decades, Jordan has undergone rapid demographic transitions characterized by accelerated population growth, urbanization, and recurring refugee inflows [1]. Beyond natural growth, the country has absorbed successive waves of displaced populations forced out by wars, mass atrocities, ethnic cleansing, and large‐scale human rights violations in Palestine, Iraq, and Syria, profoundly altering its demographic composition. Approximately 3 million refugees, including over 600,000 from Syria, currently reside in Jordan, many of whom have experienced significant trauma. In particular, large refugee influxes followed the Iraq war and the Syrian civil conflict, placing additional pressure on housing, infrastructure, and essential public services [2, 3]. Urban areas—particularly Amman—have expanded rapidly, with the urbanization rate rising from 44% in 1961 to over 83% by 2013 [4]. This growth has often occurred at the expense of rural and agricultural lands, contributing to infrastructure strain and environmental challenges, especially in water‐scarce regions [5, 6]. Refugee camps, such as Irbid camp with approximately 29,000 inhabitants, have required targeted interventions to mitigate socioeconomic pressures and promote integration [7, 8].

These demographic and geopolitical pressures have profoundly shaped Jordan's health system, which faces the dual challenge of accommodating refugee health needs while managing a rising burden of noncommunicable diseases (NCDs) [9]. Syrian refugees, in particular, face barriers to accessing chronic disease and mental health care, further straining public services [10, 11]. Meanwhile, national trends reflect growing rates of obesity, diabetes, and cardiovascular conditions, driven by a broader nutrition and lifestyle transition [12]. Nonetheless, strategic investments in cancer care, reproductive health, and preventive services have led to measurable gains, supported by institutions like UNRWA and government‐led health reforms [13, 14]. Expanded vaccination coverage and a shift toward primary care have further contributed to improving health outcomes. Yet despite these advances, Jordan lacks a comprehensive, longitudinal synthesis of disease burden that integrates and contextualizes these evolving trends. A systematic analysis of health outcomes and risk factors spanning several decades is needed to inform national priorities, promote health equity, and guide effective policy formulation.

Parallel to these demographic shifts, Jordan's socioeconomic landscape has also evolved, shaped by rising education levels, changing employment structures, and widening urban–rural disparities. Economic pressures and regional instability have affected living conditions and access to care, with households in lower‐income and refugee‐dense areas facing greater exposure to chronic disease risks and limited access to preventive services. These intersecting demographic and socioeconomic transitions have influenced lifestyle behaviors, healthcare utilization, and ultimately the distribution of disease burden across the population.

To evaluate these trends with consistency and comparability, this study employs the Global Burden of Disease (GBD) framework, developed by the Institute for Health Metrics and Evaluation (IHME). The GBD provides standardized estimates of morbidity, mortality, and risk factors across countries and over time, enabling a comprehensive assessment of national health trends. This approach has been successfully applied in numerous national and regional analyses to track progress and inform health policy. The GBD framework has been successfully applied to track national health trends and inform policy in countries such as Saudi Arabia, Pakistan, and Egypt [15, 16]. While Jordan has been featured in regional estimates and recent condition‐specific analyses, such as those focused on mental disorders [2] and cardiovascular diseases [17], a unified, all‐cause, longitudinal assessment of disease burden remains lacking.

Given Jordan's evolving demographic dynamics, shifting disease patterns, and sustained pressures from regional instability and displacement [18], understanding long‐term health trends is increasingly critical. Despite the availability of targeted studies and condition‐specific insights, a unified, longitudinal synthesis of national health outcomes remains absent. To address this gap, the present study systematically analyzes the burden of diseases, injuries, and risk factors in Jordan from 1990 to 2023 using data from the Global Burden of Disease Study. Specifically, it quantifies long‐term trends in mortality, morbidity, and disability; identifies the leading causes and risk factors contributing to overall health loss; and examines variations by age and sex. By providing a comprehensive and comparable assessment of health loss over three decades, this analysis aims to inform national public health priorities, guide evidence‐based policymaking, and support ongoing efforts to strengthen the country's health system.

## 2 Methods

### 2.1 Overview and Study Design

This study presents a comprehensive assessment of the health status and trends in Jordan from 1990 to 2023 using data from the GBD 2023 study. We employed a systematic analytical framework to estimate the burden of diseases, injuries, and risk factors across all age groups and sexes. The study was designed as a descriptive, ecological analysis based on secondary data, following the established GBD methodology developed by the IHME (ref). All results are presented at the national level, with stratifications by age and sex, and grouped into three broad cause categories: communicable, maternal, neonatal, and nutritional disorders (CMNN); non‐communicable diseases (NCDs); and injuries. The primary objective of this analysis was to examine temporal patterns in key health metrics—including mortality, prevalence, incidence, years lived with disability (YLDs), years of life lost (YLLs), and disability‐adjusted life years (DALYs)—and to evaluate shifts in disease burden over time. The study also assessed changes in the distribution of disease burden by sex and across age groups to better understand demographic and epidemiological transitions in Jordan over the past three decades.

### 2.2 Data Sources

This study used publicly available estimates from the GBD 2023 study. Estimates for Jordan were downloaded for the period from 1990 to 2023 according to the measures, years, age groups, sex categories, causes, and risk factors required for the analysis. The GBD estimates are informed by several sources, including civil registration and vital statistics systems, population censuses, household surveys, health facility records, and disease registries. The data were collected and modeled by the IHME as part of the wider GBD study. This study did not involve access to or reanalysis of the individual records used to produce the estimates. Further information on the sources used for Jordan is available through the GBD Sources Tool (http://ghdx.healthdata.org/gbd-2023/data-input-sources).

### 2.3 Metrics and Definitions

The health metrics examined in this study were mortality, prevalence, YLLs, YLDs, and DALYs. In the GBD framework, DALYs are calculated as the sum of YLLs and YLDs and represent health loss resulting from both premature mortality and non‐fatal conditions. YLLs are based on the number of deaths at each age and the remaining standard life expectancy at that age. YLDs are based on condition prevalence and the corresponding disability weights, with adjustment for comorbidity during the GBD estimation process.

Estimates are presented as age‐standardized rates per 100,000 population using the GBD world standard population, unless otherwise specified. Results were disaggregated by age group, sex, and year, and categorized into three broad cause groups: CMNN, NCDs, and injuries. For the principal cause rankings, we used mutually exclusive GBD Level 3 causes to avoid ranking parent categories alongside their component conditions. Level 1 categories were used only for the broad CMNN, NCD, and injury summaries in Figures S1A, S1B, S1C and S2A, S2B, S2C. Level 2 cause groups were used to show the distribution of risk‐attributable burden in Figure 5A,B. Risk factor rankings were restricted to terminal risks in the GBD hierarchy.

Risk factors were grouped by behavioral, metabolic, and environmental/occupational domains, and their attributable burden was assessed in terms of both DALYs and deaths. Risk‐attributable estimates were obtained from the GBD 2023 study and were not independently modeled as part of this analysis.

Supporting Information S2: Table S1 provides the definitions and hierarchical classifications of the causes, risk factors, measures, and analytical terms included in the analysis.

### 2.4 Statistical Modeling

The GBD 2023 study uses a range of established models to estimate fatal and non‐fatal health outcomes. These include the Cause of Death Ensemble model (CODEm) for causes of death and DisMod‐MR for non‐fatal disease outcomes. Procedures such as garbage‐code redistribution, age‐sex splitting, covariate adjustment, and uncertainty estimation were carried out by the IHME when producing the GBD estimates and were not repeated in the present study. Further details are available in the GBD technical documentation.

The GBD estimates incorporate standard procedures for addressing limitations in the underlying data, including garbage‐code redistribution, age‐sex splitting, and covariate adjustment. Estimates were accompanied by 95% uncertainty intervals derived from 1000 posterior draws, reflecting uncertainty in the data inputs and modeling procedures. For the present study, the downloaded GBD estimates were processed, summarized, and visualized using R (version 4.3.2). Further details of the methods used to generate the GBD estimates are available through the GBD technical appendices [19].

### 2.5 Ethics and Data Availability

This analysis used publicly available, de‐identified data from the GBD 2023 study and did not require ethical approval. All estimates are accessible through the GBD Results Tool (http://ghdx.healthdata.org/gbd-results-tool) and the GBD Compare visualization platform (https://vizhub.healthdata.org/gbd-compare/).

## 3 Results

### 3.1 Overall Trends

From 1990 to 2023, Jordan recorded a 33.88% (95% UI: −37.38 to −30.59) decline in age‐standardized DALYs for both sexes combined, with males showing a −38.83% (−42.22 to −35.60) reduction and females −27.22% (−31.33 to −23.58) (Table 1, Figure 1). YLLs decreased by −49.38% (−50.93 to −47.68) overall, with males (−51.85%; −53.74 to −49.81) again showing greater reductions than females (−45.79%; −48.29 to −43.15). In contrast, YLDs changed by 2.49% (−2.37 to 7.47) overall, with the uncertainty interval including no change. The all‐cause point‐prevalence rate declined modestly by −1.34% (−2.13 to −0.62) over the 33‐year period. Although males continued to show higher DALY rates than females, the difference narrowed from approximately 7305 per 100,000 in 1990 to 515 per 100,000 in 2023. The annual distributions of the three broad GBD cause categories for prevalence, deaths, and DALYs are shown in Figures S1A, S1B and S1C, respectively. The corresponding all‐age rates for the three cause categories in 2023 are shown in Figures S2A, S2B, and S2C.

**Table 1:** Percentage change in age‐standardized all‐cause burden rates in Jordan across 1990–2000, 2000–2010, 2010–2023, and 1990–2023.

Measure	Sex	1990–2000 (Age‐standardized)	2000–2010 (Age‐standardized)	2010–2023 (Age‐standardized)	1990–2023 (Age‐standardized)
Deaths	Male	−8.56% (−10.92 to −5.81)	−19.68% (−21.72 to −17.35)	−21.13% (−24.72 to −17.20)	−42.07% (−44.93 to −39.14)
	Female	3.92% (0.64 to 7.20)	−16.39% (−18.77 to −13.78)	−25.08% (−29.79 to −20.09)	−34.91% (−39.14 to −30.92)
	Both	−3.75% (−5.80 to −1.53)	−18.18% (−19.75 to −16.49)	−22.65% (−25.49 to −19.51)	−39.08% (−41.62 to −36.58)
DALYs	Male	−8.86% (−10.81 to −7.12)	−17.86% (−19.73 to −16.06)	−18.29% (−21.38 to −14.93)	−38.83% (−42.22 to −35.60)
	Female	−1.45% (−3.67 to 1.03)	−13.13% (−15.30 to −10.92)	−14.99% (−18.55 to −11.82)	−27.22% (−31.33 to −23.58)
	Both	−5.66% (−7.12 to −4.16)	−15.71% (−17.54 to −14.06)	−16.85% (−19.93 to −13.97)	−33.88% (−37.38 to −30.59)
YLDs	Male	−0.26% (−4.03 to 3.04)	0.42% (−3.38 to 3.58)	3.97% (−0.02 to 8.24)	4.13% (−2.17 to 9.30)
	Female	−1.64% (−5.11 to 2.11)	0.03% (−2.99 to 3.16)	4.06% (0.91 to 7.81)	2.39% (−2.78 to 8.05)
	Both	−0.80% (−4.34 to 2.33)	−0.04% (−3.07 to 2.57)	3.36% (0.03 to 7.03)	2.49% (−2.37 to 7.47)
YLLs	Male	−11.46% (−13.48 to −9.46)	−24.10% (−25.71 to −22.47)	−28.35% (−31.13 to −25.33)	−51.85% (−53.74 to −49.81)
	Female	−1.33% (−3.94 to 1.49)	−21.36% (−23.30 to −19.36)	−30.14% (−33.73 to −26.41)	−45.79% (−48.29 to −43.15)
	Both	−7.72% (−9.24 to −6.08)	−22.90% (−24.20 to −21.64)	−28.85% (−31.07 to −26.50)	−49.38% (−50.93 to −47.68)
Prevalence	Male	−0.61% (−1.35 to 0.13)	−0.95% (−1.60 to −0.36)	−0.22% (−1.02 to 0.50)	−1.77% (−2.84 to −0.79)
	Female	−0.32% (−0.70 to −0.03)	−0.44% (−0.77 to −0.17)	−0.11% (−0.51 to 0.33)	−0.87% (−1.40 to −0.39)
	Both	−0.46% (−0.96 to 0.02)	−0.72% (−1.15 to −0.34)	−0.17% (−0.77 to 0.36)	−1.34% (−2.13 to −0.62)

*Note*. Values are percentage changes with 95% uncertainty intervals from the GBD direct percentage‐change extract. The all‐cause prevalence row is not the arithmetic sum of cause‐specific prevalence estimates.

**Figure 1:**
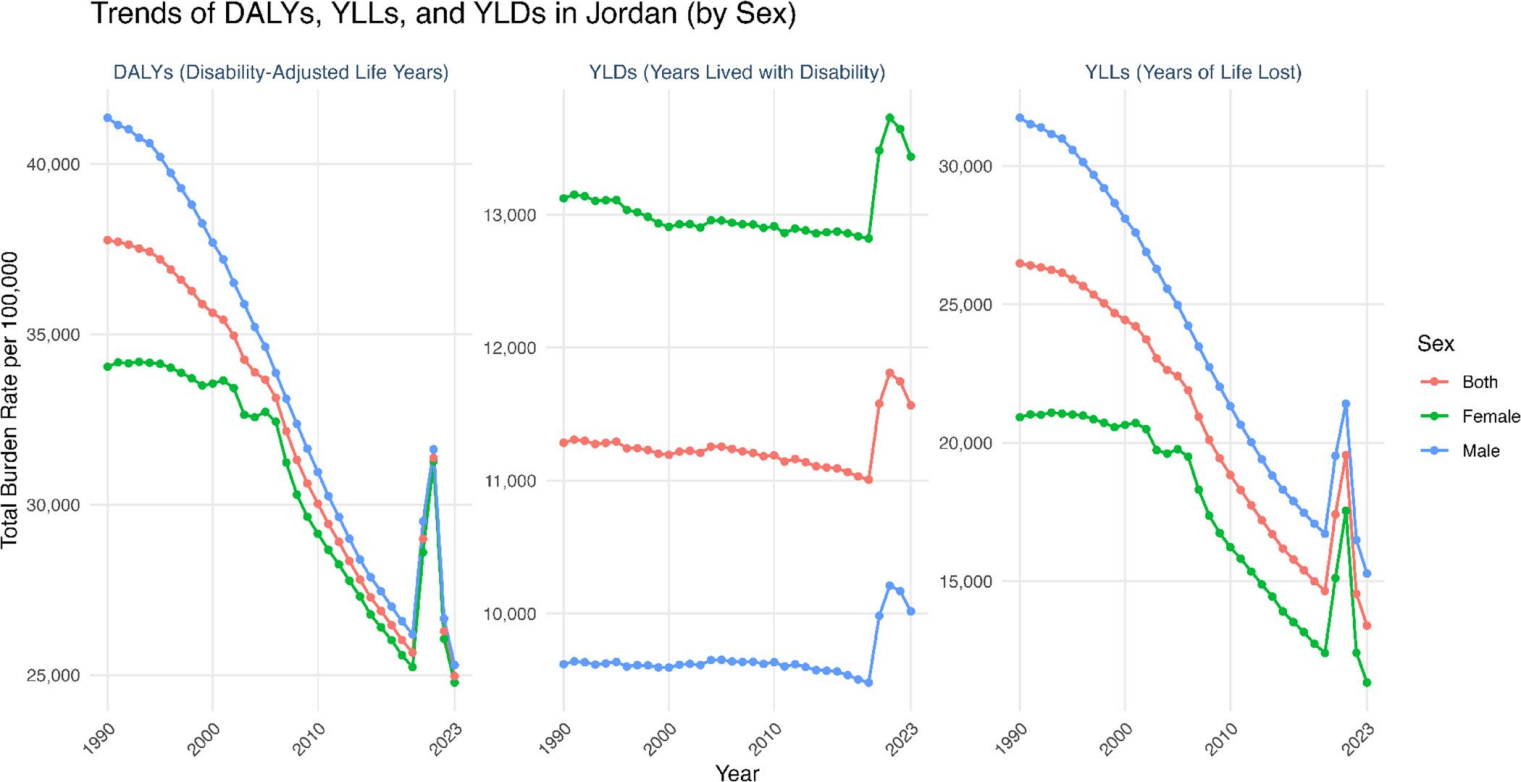
Trends in age‐standardized DALY, YLL, and YLD rates in Jordan by sex, 1990–2023. *Note:* Rates are per 100,000 population. DALYs equal YLLs plus YLDs. The corrected panels use the source measure corresponding to each y‐axis.

### 3.2 Leading Causes of Burden

Between 1990 and 2023, the burden of disease in Jordan shifted from neonatal and communicable conditions toward NCDs as measured by DALYs (Table 2, Figure 2). Using mutually exclusive GBD Level 3 causes ranked by all‐age DALY rates, neonatal disorders, the leading cause of DALYs in 1990, fell to 7th place by 2023. In contrast, ischemic heart disease rose from 4th to 1st place, while diabetes mellitus rose from 7th to 2nd. Congenital birth defects moved from 2nd to 3rd, stroke from 5th to 4th, and depressive disorders from 13th to 5th. Road injuries declined from 6th to 10th place. These all‐age rankings should be considered alongside the changes in age‐standardized rates, which account for differences in population age structure over time.

**Table 2:** Top 20 level 3 causes of DALYs in Jordan in 1990, 2000, 2010, and 2023.

Leading cause 1990	All‐age DALY rate 1990	Leading cause 2000	All‐age DALY rate 2000	Leading cause 2010	All‐age DALY rate 2010	Leading cause 2023	All‐age DALY rate 2023
Neonatal disorders	5224.02 (4674.77–5767.41)	Neonatal disorders	4350.50 (3821.87– 4792.88)	Neonatal disorders	2567.85 (2384.67– 2758.06)	Ischemic heart disease	1439.92 (1215.61– 1638.39)
Congenital birth defects	2869.71 (2102.48– 3571.37)	Congenital birth defects	2803.81 (2211.00– 3458.74)	Congenital birth defects	2054.95 (1780.42– 2324.58)	Diabetes mellitus	1144.80 (865.27– 1439.64)
Lower respiratory infections	2075.57 (1570.46– 2697.34)	Ischemic heart disease	1763.73 (1477.42– 2024.81)	Ischemic heart disease	1423.94 (1323.47– 1537.35)	Congenital birth defects	981.12 (847.98– 1114.87)
Ischemic heart disease	1827.86 (1368.91– 2275.45)	Stroke	1539.92 (1272.27– 1907.69)	Stroke	1029.20 (922.18– 1149.36)	Stroke	918.30 (771.41– 1088.85)
Stroke	1622.55 (1185.60– 2109.77)	Lower respiratory infections	1409.86 (1114.43– 1725.69)	Diabetes mellitus	970.04 (801.58– 1160.50)	Depressive disorders	877.49 (589.02– 1289.61)
Road injuries	1607.79 (1139.78– 2108.68)	Road injuries	1192.90 (940.70– 1475.01)	Road injuries	914.79 (769.83– 1056.18)	Low back pain	860.32 (606.43– 1152.98)
Diabetes mellitus	720.39 (572.10– 875.91)	Diabetes mellitus	863.49 (702.42– 1019.63)	Lower respiratory infections	853.64 (731.09– 996.63)	Neonatal disorders	854.22 (718.65– 984.95)
Dietary iron deficiency	717.53 (312.65– 1410.05)	Low back pain	699.31 (493.38– 939.94)	Low back pain	743.71 (519.82– 1007.45)	Anxiety disorders	704.55 (476.24– 1040.19)
Low back pain	648.86 (445.91– 868.65)	Dietary iron deficiency	627.72 (325.41– 1146.64)	Depressive disorders	579.15 (396.09– 800.54)	Headache disorders	623.36 (425.24– 864.65)
Headache disorders	523.20 (355.13– 730.46)	Headache disorders	555.14 (374.46– 754.90)	Headache disorders	573.67 (389.13– 793.54)	Road injuries	617.17 (485.10– 764.99)
Anxiety disorders	522.10 (317.08– 809.15)	Anxiety disorders	520.79 (336.82– 758.93)	Anxiety disorders	510.31 (335.80– 721.28)	Chronic kidney disease	587.67 (488.48– 701.57)
Dermatitis	484.38 (258.99– 834.86)	Depressive disorders	518.76 (342.86– 720.56)	Dietary iron deficiency	507.27 (305.96– 848.42)	Gynecological diseases	495.45 (350.39– 697.83)
Depressive disorders	471.12 (305.31–707.99)	Dermatitis	456.06 (241.40– 790.80)	Gynecological diseases	461.56 (322.19– 647.67)	Dietary iron deficiency	454.21 (272.68– 736.84)
Chronic kidney disease	437.46 (316.51– 578.53)	Chronic kidney disease	446.63 (334.90– 566.20)	Chronic kidney disease	449.56 (388.01– 512.40)	Other musculoskeletal disorders	448.61 (306.95– 617.95)
Gynecological diseases	376.13 (263.06– 528.91)	Gynecological diseases	428.31 (298.93– 602.33)	Dermatitis	435.51 (235.89– 757.45)	Hypertensive heart disease	431.12 (334.07– 526.51)
Falls	345.80 (269.71– 457.93)	Hypertensive heart disease	345.07 (262.98– 451.46)	Hypertensive heart disease	377.95 (294.50– 441.86)	Lower respiratory infections	424.08 (350.86– 513.37)
Leukemia	338.57 (258.88– 440.74)	Falls	322.19 (254.43– 402.98)	Other musculoskeletal disorders	356.65 (254.61– 491.60)	Age‐related and other hearing loss	401.09 (280.81– 535.15)
Hypertensive heart disease	324.22 (207.45– 458.48)	Leukemia	312.53 (253.99– 380.01)	Falls	329.08 (266.04– 411.69)	Dermatitis	377.65 (205.87– 639.99)
Age‐related and other hearing loss	290.84 (202.15– 383.50)	Diarrheal diseases	308.85 (198.48– 478.04)	Age‐related and other hearing loss	323.44 (224.95– 430.32)	COVID‐19	354.10 (211.12– 588.10)
Diarrheal diseases	285.44 (169.06– 444.09)	Age‐related and other hearing loss	306.81 (213.34– 405.80)	Interpersonal violence	272.46 (233.72– 310.43)	Neck pain	326.84 (217.85– 489.98)

*Note*. Rankings are independently ordered for each year using all‐age DALY rates per 100,000 for both sexes. All causes are mutually exclusive. Level 3 nodes and values include 95% uncertainty intervals.

**Figure 2:**
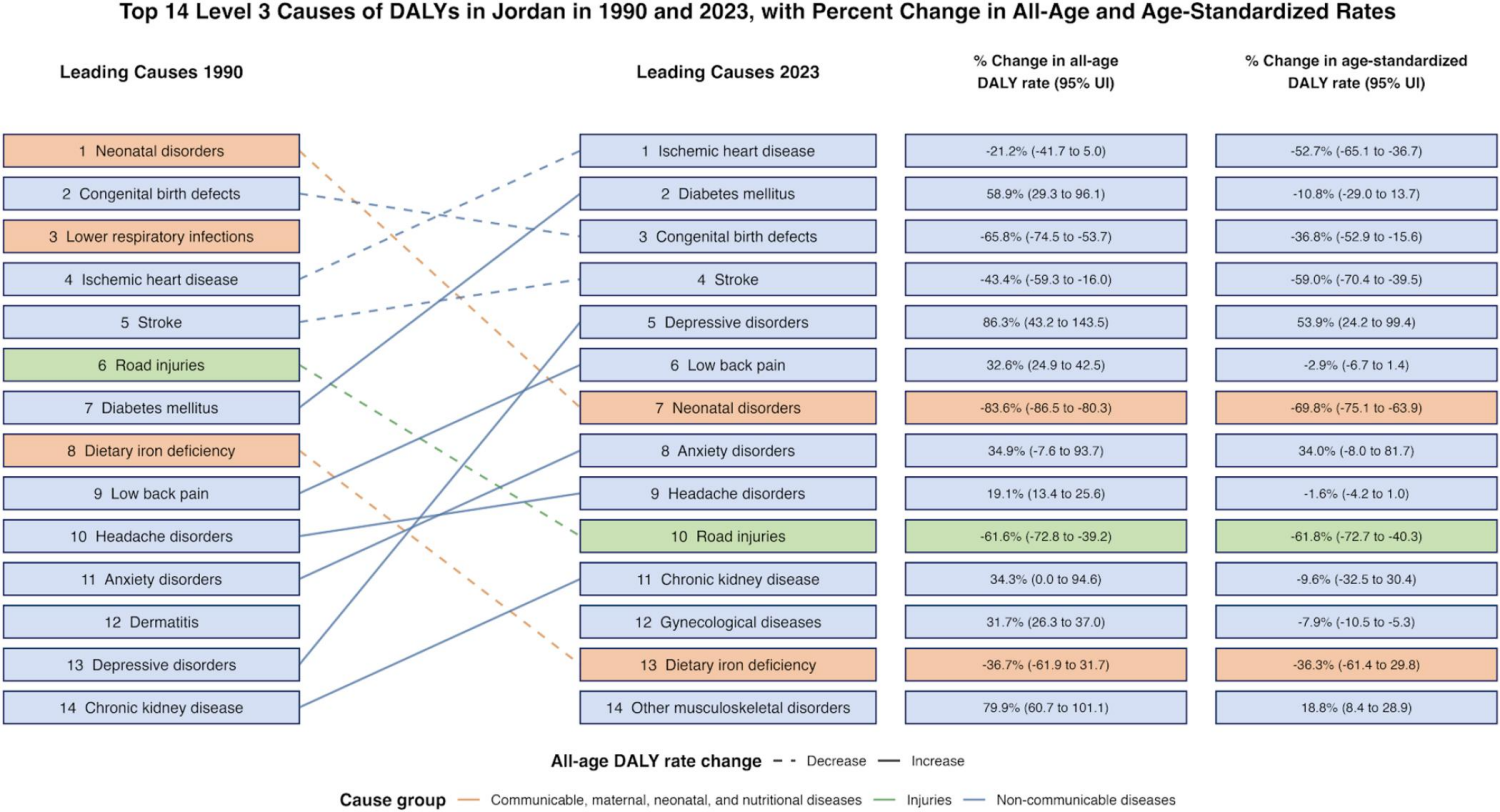
Top 14 mutually exclusive Level 3 causes of DALYs in Jordan in 1990 and 2023, with changes in all‐age and age‐standardized rates. *Note:* Rankings use all‐age DALY rates for both sexes. Solid connecting lines indicate an increase and dashed lines indicate a decrease in the all‐age DALY rate. Percentage changes include 95% uncertainty intervals.

### 3.3 Age Differences

Disease burden in Jordan varied substantially by age group in 2023. The leading causes of DALYs and deaths across non‐overlapping age groups are shown in Figure 3a,b, respectively. Neonatal disorders and congenital birth defects showed the highest DALY rates during the earliest stages of life. Among children, congenital birth defects, dermatitis, road injuries, and dietary iron deficiency were prominent causes of burden. Adolescents showed a transition toward mental disorders, with anxiety disorders ranking highest among those aged 10–14 years and depressive disorders ranking highest among those aged 15–19 years. Road injuries also remained an important cause of burden, particularly for deaths among older adolescents and young adults. The age composition of the leading causes of prevalence, deaths, and DALYs, using non‐overlapping age groups and total numbers, is presented in Figures S3A, S3B, and S3C, respectively.

**Figure 3:**
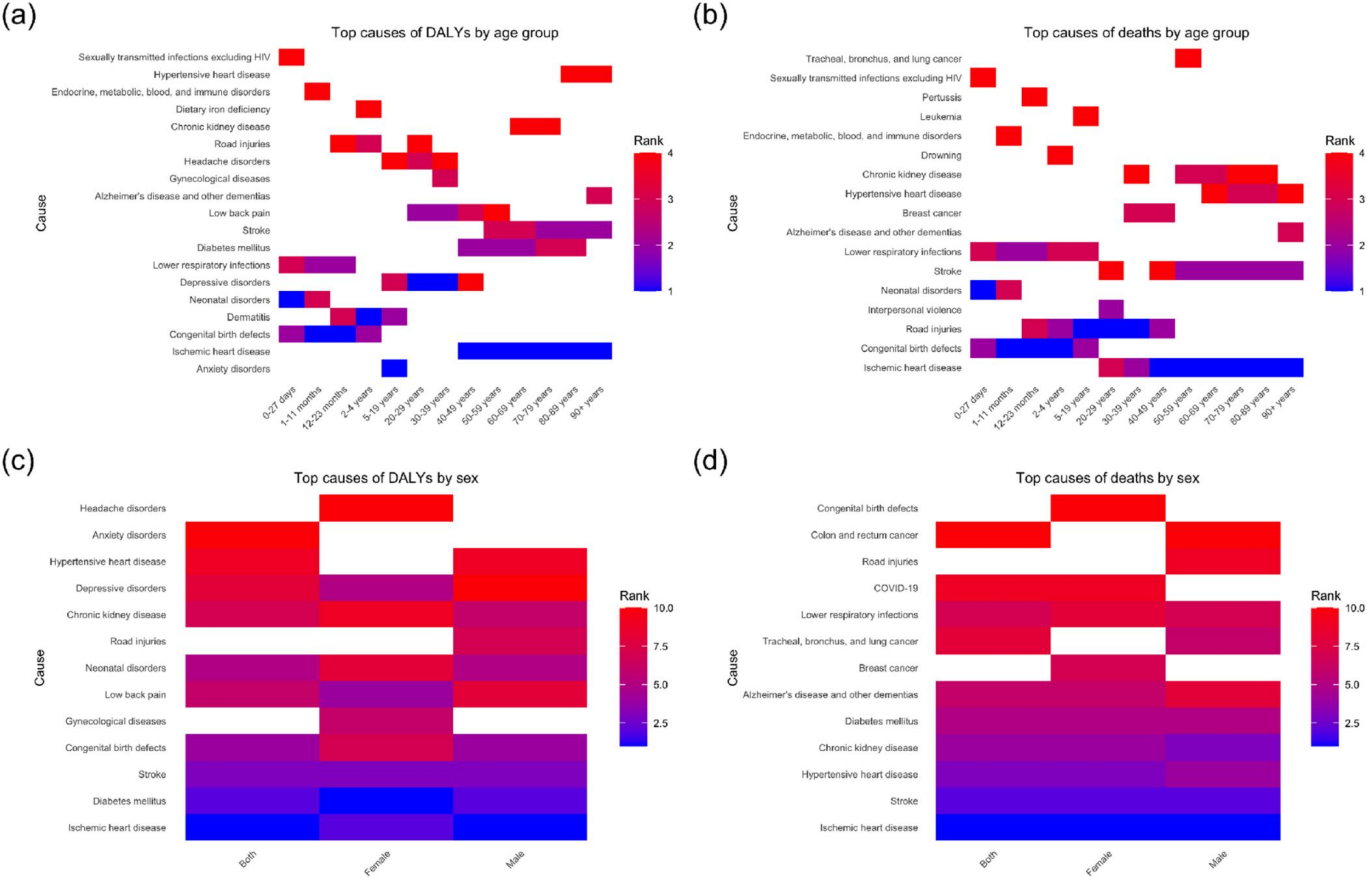
Leading level 3 causes of DALYs and deaths in Jordan by non‐overlapping age group and sex, 2023. (a) Leading causes of DALYs by age group; (b) leading causes of deaths by age group; (c) leading causes of DALYs by sex; and (d) leading causes of deaths by sex. *Note:* Age‐specific panels display the four leading causes in each grouped age band. Sex‐specific panels display the 10 leading age‐standardized causes for both sexes, females, and males. Blank cells mean that a cause was not within the displayed top causes, not that data were missing.

### 3.4 Gender Differences

In 2023, sex‐specific disease burden patterns were evident for both DALYs and mortality, as shown in Figure 3c,d, respectively. For DALYs, males showed the highest burden from ischemic heart disease, followed by diabetes mellitus and stroke, while females showed the highest burden from diabetes mellitus, ischemic heart disease, and stroke. Mortality patterns were similar, with ischemic heart disease ranking first for both males and females. Road injuries appeared among the top 10 causes of DALYs and deaths for males, but not for females. These findings show differences in the distribution of disease burden by sex, although the present analysis was not designed to determine the mechanisms underlying these patterns.

### 3.5 Regional and Global Comparisons

Jordan's age‐standardized DALY rates for selected mutually exclusive GBD Level 3 causes were compared with estimates for the Eastern Mediterranean Region (EMR), the Middle East and North Africa (MENA) region, and globally in Figure 4A. In 2023, the DALY rate for ischemic heart disease in Jordan was substantially lower than the EMR and MENA rates and was close to the global rate. In contrast, the DALY rate for diabetes mellitus in Jordan was higher than all three comparator estimates and was approximately 1.56 times the global rate. Jordan's stroke DALY rate was lower than the corresponding EMR, MENA, and global rates. The DALY rates for hypertensive heart disease and chronic kidney disease were higher in Jordan than the global estimates and were broadly comparable to or higher than the regional estimates. Road injuries and neonatal disorders had lower DALY rates in Jordan than in each of the three comparator populations. Lower respiratory infections also showed a lower DALY rate in Jordan than the regional and global estimates. Corresponding age‐standardized death rates for the selected causes are presented in Figure 4B.

**Figure 4:**
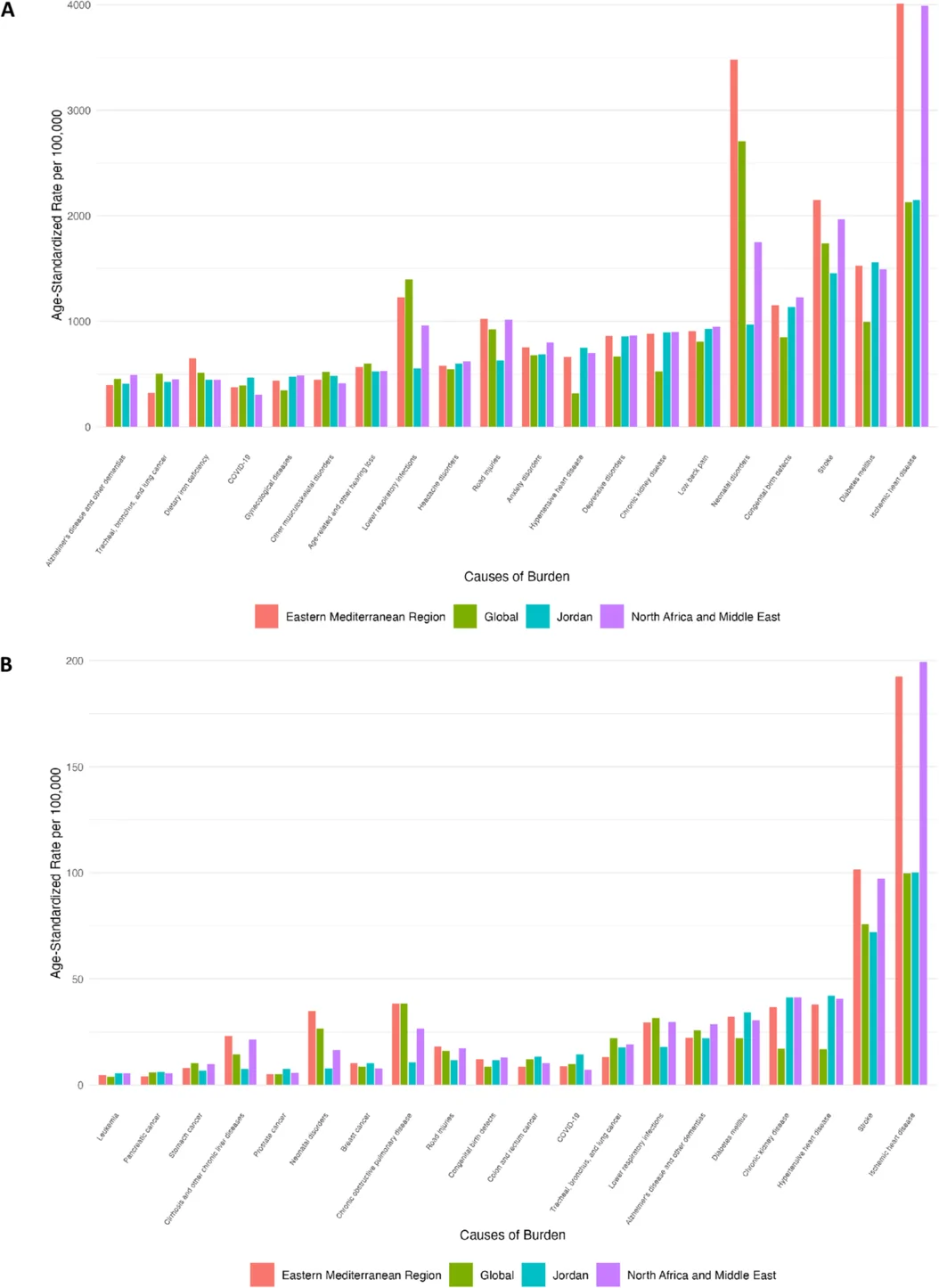
(A) Comparison of age‐standardized DALY rates for Jordan's 20 leading Level 3 DALY causes with regional and global estimates, 2023. *Note:* Causes were selected independently using Jordan's DALY ranking. Comparator values are GBD aggregate estimates, not simple arithmetic averages. (B) Comparison of age‐standardized death rates for Jordan's 20 leading Level 3 causes of death with regional and global estimates, 2023. *Note:* Causes were selected independently using Jordan's death ranking; consequently, the cause set differs from panel A. Comparator values are GBD aggregate estimates.

### 3.6 Risk Factors

Leading risk factors and their percentage changes in contributions to DALYs and deaths between 1990 and 2023 are shown in Table 3. Figure 5A,B present the leading risk factors contributing to age‐standardized DALY and death rates in 2023, respectively. High BMI was the leading contributor to age‐standardized DALYs, followed by high fasting plasma glucose, high systolic blood pressure, and smoking. High BMI accounted for approximately 11.5% of total DALYs in 2023. Environmental and metabolic exposures, including ambient particulate matter pollution and kidney dysfunction, also contributed to the overall burden. Because individuals may be exposed to multiple risks, risk‐attributable burdens overlap and should not be summed across risk factors. Environmental exposures, including ambient particulate matter pollution and household air pollution from solid fuels, also featured prominently.

**Table 3:** Percentage change in burden attributable to the leading leaf risk factors in Jordan between 1990 and 2023.

Risk factor	% Change in DALYs (all‐age)	% Change in DALYs (ASR)	% Change in deaths (all‐age)	% Change in deaths (ASR)
High body‐mass index	53.92%	−10.03%	40.10%	−14.33%
High fasting plasma glucose	46.75%	−17.06%	24.48%	−26.25%
High systolic blood pressure	−6.58%	−44.90%	0.31%	−40.75%
Smoking	−1.88%	−44.79%	−9.25%	−47.29%
Ambient particulate matter pollution	−37.32%	−33.42%	−5.53%	−24.95%
Kidney dysfunction	7.95%	−32.64%	18.50%	−29.12%
High LDL cholesterol	−33.60%	−61.23%	−30.12%	−58.49%
Low birth weight	−84.63%	−71.04%	−88.20%	−75.97%
Short gestation	−84.06%	−70.48%	−88.49%	−76.59%
Secondhand smoke	−40.59%	−49.08%	−26.03%	−48.62%
Diet low in fruits	26.54%	−22.22%	35.74%	−16.33%
Diet low in whole grains	−4.60%	−43.92%	−6.49%	−43.96%
Iron deficiency	−37.72%	−37.64%	−76.47%	−81.67%
Low temperature	−45.34%	−57.84%	−25.59%	−52.20%
Diet low in vegetables	6.26%	−35.07%	18.99%	−27.95%

*Note*. Risks are terminal nodes selected by their 2023 age‐standardized DALY rate. All‐age and age‐standardized changes are shown for deaths and DALYs. Risk burdens overlap and should not be summed across risk factors.

**Figure 5:**
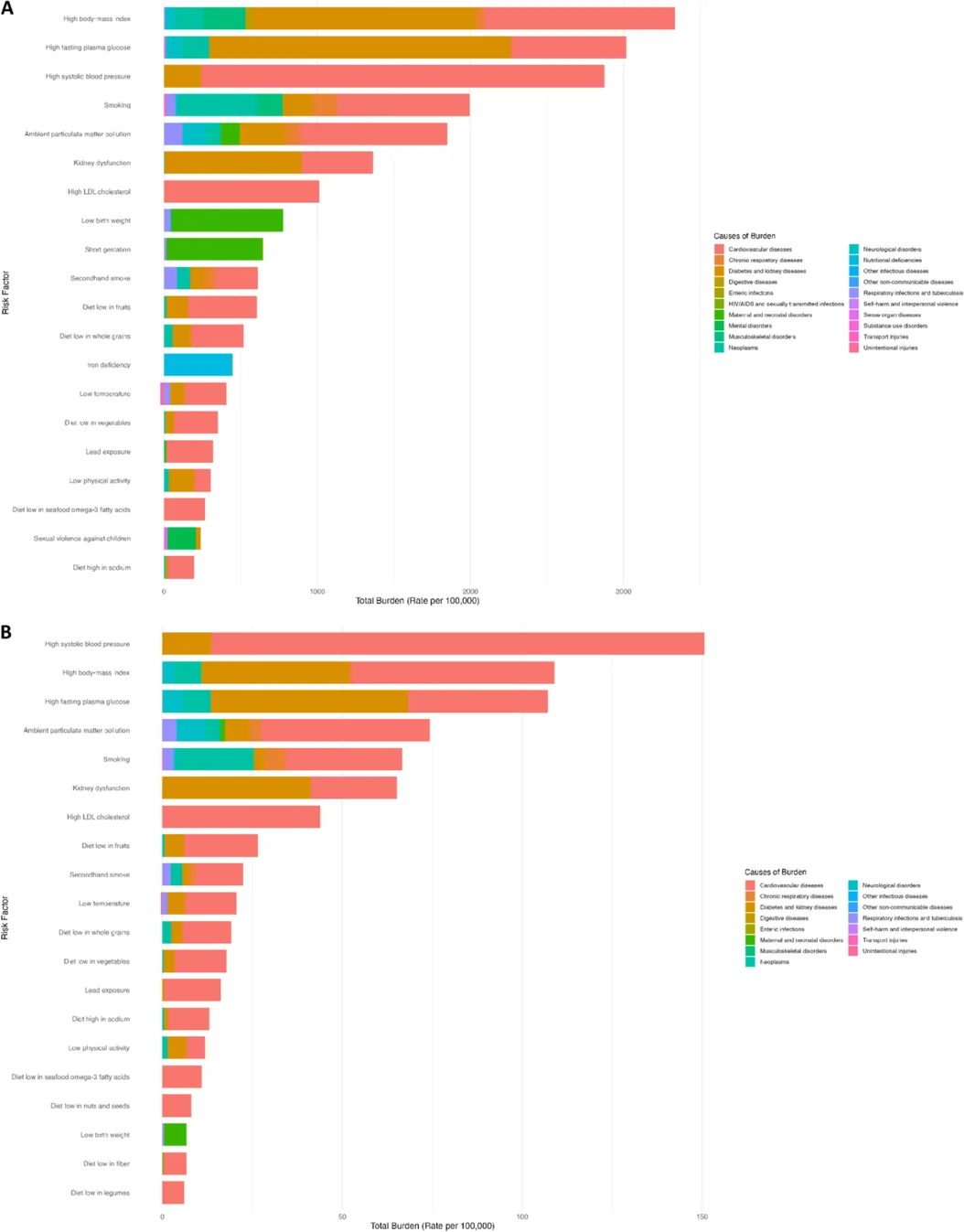
(A) Leading leaf risk factors contributing to age‐standardized DALY rates in Jordan, 2023, decomposed by Level 2 cause group. *Note:* Risk factors are terminal nodes in the GBD risk hierarchy and were selected independently for DALYs. Attributable burdens overlap across risk factors and must not be summed across rows. (B) Leading leaf risk factors contributing to age‐standardized death rates in Jordan, 2023, decomposed by Level 2 cause group. *Note:* Risk factors were selected independently for deaths. Attributable burdens overlap across risk factors and must not be summed across rows.

In 2023, mortality in Jordan was predominantly driven by modifiable metabolic and behavioral risk factors (Figure 5B). The top cause of risk‐attributable deaths was high systolic blood pressure, followed closely by high fasting plasma glucose and high BMI. Tobacco use was the fifth leading contributor to mortality. While kidney dysfunction was less prominent than in DALY rankings, they still contributed notably to the overall mortality profile.

## 4 Discussion

This study provides the first comprehensive, longitudinal analysis of the burden of disease, risk factors, and mortality trends in Jordan from 1990 to 2023, using the GBD framework. Despite the availability of condition‐specific studies and regional assessments, a unified, longitudinal synthesis of national health outcomes in Jordan has not been previously conducted. This study addresses that gap by analyzing disease, injury, and risk factor trends in Jordan from 1990 to 2023 using GBD data. Previous GBD‐based work, most notably Mokdad et al.'s 2014 analysis, grouped Jordan with other middle‐income Arab countries and included data only through 2010 [20]. Since then, Jordan has undergone substantial demographic, epidemiologic, and health system transitions that merit focused national analysis [17]. Between 1990 and 2023, Jordan saw a 33.9% decline in age‐standardized DALYs, driven largely by reductions in YLLs, especially among males. In contrast, YLDs remained relatively stable, with modest increases after 2020. The burden of disease shifted from CMNN conditions to NCDs such as type 2 diabetes and low back pain. Sex differences persisted throughout, with males bearing higher DALYs and YLLs, and a widening gender gap in YLDs in recent years—highlighting an aging population increasingly impacted by chronic illness and disability.

The sharp decline in YLLs (nearly 50%) points to broad gains in survival. Improvements in neonatal and pediatric care likely played a key role, as shown by the declines in neonatal preterm birth and congenital heart anomalies. These changes reflect investments in maternal‐child health programs, vaccinations, and primary care [21]. Yet, regional disparities remain: southern hospitals have more incubators, while northern ones are better equipped with CPAP, phototherapy, and resuscitation equipment [22]—highlighting the need for equitable infrastructure scaling. Road injury burden also declined substantially over the study period, with the age‐standardized DALY rate for road injuries decreasing by approximately 61.7% between 1990 and 2023. This decline occurred alongside the implementation of road safety legislation, strengthened traffic enforcement, and the development of a national trauma system incorporating measures such as Advanced Trauma Life Support training and improvements in prehospital trauma care [23, 24]. Although these developments may have contributed to improved outcomes, the present analysis did not assess their causal effects.

Furthermore, pediatric care continues to evolve, with recent efforts to scale up national strategies for cancer and chronic disease management in children [25].

However, the burden of nonfatal disability remained comparatively stable. The age‐standardized YLD rate changed by 2.49% between 1990 and 2023, with an uncertainty interval that included no change. Nonetheless, several chronic conditions became more prominent in the all‐age DALY rankings. Diabetes mellitus rose from 7th to 2nd place, while low back pain rose from 9th to 6th. The increasing prominence of diabetes in the all‐age ranking, despite no clear increase in its age‐standardized DALY rate, suggests that population growth and aging contributed to the expanding absolute burden of the condition. The growing prevalence of NCDs reflects underlying drivers such as obesity, sedentary lifestyles, and dietary shifts, highlighting the need for primordial prevention to curb the emergence of these risks. Jordan's obesity rates are among the highest in the region [26], affecting over half of adult women and a third of men. Urbanization has fueled increased consumption of ultra‐processed foods and reduced physical activity, with similar trends observed across MENA [27]. Greater availability of inexpensive imported processed foods and persistent socioeconomic constraints also shape unhealthy dietary patterns, making sedentary lifestyles and low physical activity key modifiable drivers of metabolic and musculoskeletal disease [28].

Sex‐disaggregated patterns further demonstrated differences in the distribution of disease burden. Ischemic heart disease was the leading cause of DALYs among males, while diabetes mellitus ranked first among females. Ischemic heart disease was the leading cause of death in both sexes. Road injuries appeared among the top 10 causes of DALYs and deaths for males, but not for females. Notably, there has been prior work published in the literature exemplifying that men are less likely to seek Timely care due to gender norms that equate help‐seeking with weakness, compounded by stigma around mental health and competing economic pressures in the region [29, 30, 31, 32]. However, the present analysis did not directly assess the behavioral, biological, cultural, or health‐system factors contributing to these differences.

While biological and systemic factors both contribute, barriers such as reduced health autonomy, cost, geographic access, and discrimination still hinder many women's ability to receive timely care [33, 34, 35]. Lower decision‐making power within households, financial dependence on male family members, and cultural norms requiring spousal or family permission for care often restrict women's ability to seek services promptly [36, 37]. Among children, the shift from infectious to chronic, non‐fatal conditions—particularly dermatologic disorders—may be driven by both improved survival and reduced caregiver recognition of non‐lethal health needs [38].

Finally, the transition in risk factors reflects the broader epidemiological shift. In 2023, high BMI, systolic blood pressure, and fasting plasma glucose ranked as the top three drivers of DALYs, signaling a dominant metabolic profile. Obesity in Jordan is widespread, especially among women and children, driven by lifestyle and environmental changes [26, 39]. While health promotion efforts and weight loss intervention programs exist across Jordanian communities, structural interventions remain fragmented [40, 41]. Tobacco use—especially among men aged 25–34—remains a major concern despite national control strategies [42]. Widespread waterpipe use, enforcement gaps, and limited health care provider involvement in cessation efforts continue to undermine progress [43, 44, 45]. Meanwhile, environmental exposures like ambient and household air pollution remain relevant contributors to health loss, though their share has slightly declined.

### 4.1 Policy Implications

Jordan's epidemiological transition, from early mortality due to CMNN conditions to a growing burden of chronic disease and disability, demands a realignment of public health priorities. Primordial and primary prevention efforts, delivered through coordinated multisectoral initiatives, must take center stage. National strategies should scale up obesity prevention through structural interventions, including mandatory nutrition labeling, restrictions on marketing ultra‐processed foods to children, and fiscal policies that disincentivize sugary beverage and high‐sodium food consumption. These efforts should be complemented by active living campaigns and community‐level programs that embed physical activity into daily life. Urban planning and municipal sectors should also be engaged to increase access to green spaces, enhance neighborhood walkability, and create safer environments for routine physical activity. Schools, workplaces, and municipalities must be engaged as implementation partners, not just awareness platforms. Health promotion should be accompanied by expanded access to routine risk screening in primary care—particularly for diabetes, hypertension, and liver disease—paired with longitudinal follow‐up systems to prevent silent progression of chronic conditions.

Equity must be a central pillar of any national response. The disproportionate burden of premature mortality among men—particularly from preventable cardiometabolic diseases—calls for targeted outreach programs in male‐dominated workplaces, the military, and sports clubs. Interventions should be tailored to overcome stigma around seeking care, especially for mental health and addiction. Simultaneously, persistent structural barriers that limit women's access to preventive and specialty care—including cost, transportation, autonomy, and limited facility availability in rural areas—require scalable solutions like mobile clinics, telemedicine, and community‐based women's health services. Pediatric programs must also adapt to the new burden profile: routine school‐based screenings for obesity, skin conditions, and dental disease could help identify early‐onset disability risks and ensure timely referral. Lastly, sustained investment in data infrastructure—including national registries, surveillance surveys, and implementation research—will be essential to monitor progress, evaluate interventions, and guide future recalibrations of disease burden in Jordan.

### 4.2 Strengths and Limitations

This study offers the first comprehensive, longitudinal analysis of disease burden, risk factors, and mortality trends in Jordan using GBD data spanning more than three decades. A major strength is the standardized methodology of the GBD framework, which enables robust comparisons across time, age groups, sexes, and causes. By incorporating multiple sources, including survey data, administrative records, and modeled estimates, the GBD provides a harmonized and internally consistent view of population health that captures both fatal and non‐fatal outcomes. The ability to disaggregate by age, sex, and cause further enhances the relevance of the findings for national policy planning and health system design.

However, several limitations should be acknowledged. First, the GBD relies on modeled estimates that may not fully reflect local surveillance gaps or underreporting, particularly for conditions such as COPD, mental illness, or skin diseases, which may be underdiagnosed in routine settings. Second, although the GBD incorporates multiple data sources, some inputs for Jordan may be outdated, sparse, or limited in quality—especially for subnational or risk factor–specific analyses. Third, changes in diagnostic practices and coding accuracy over time may influence observed trends, particularly for emerging conditions like MASLD. Importantly, GBD estimates cannot be stratified by country of origin, refugee status, or migrant background, limiting the ability to quantify disparities in health outcomes among the sizable displaced populations residing in Jordan. While the GBD draws on nationally representative surveys and administrative sources, coverage of refugees and migrants may be incomplete, raising questions about how well these populations are captured in underlying data streams. Lastly, although age‐standardization enables meaningful trend comparisons, it may obscure absolute shifts in burden driven by population growth and aging. Several additional limitations should be acknowledged. Cause rankings may differ according to the level of the GBD hierarchy used in the analysis. To improve consistency and avoid overlap, the principal rankings were restricted to mutually exclusive Level 3 causes, while risk factor rankings were restricted to terminal leaf risks. Risk‐attributable burdens may overlap and should not be summed across risks. Percentage‐change uncertainty intervals were obtained directly from the supplied GBD extracts. Independent reconstruction of these intervals was not possible because posterior draw files were not available. These limitations highlight the need for continued investment in national health data systems, including registries, routine surveillance, and more inclusive data infrastructure to support future GBD iterations and locally led burden of disease studies.

### 4.3 Future Research

Future research should focus on strengthening the granularity and quality of public health data in Jordan to support more precise burden estimates and localized tailored interventions. Subnational analyses—disaggregated by governorate, socioeconomic status, or refugee status—would offer valuable insights into geographic and social inequalities in health outcomes. There is also a need for primary research on the epidemiology of emerging or underrecognized conditions, including MASLD, mental health disorders, and musculoskeletal diseases, which may be underrepresented in existing surveillance systems. In parallel, implementation research evaluating the real‐world effectiveness of tobacco control, obesity prevention, and chronic disease management programs can inform policy adaptations and guide resource allocation. Finally, longitudinal cohort studies linking individual risk profiles to health outcomes are critical for improving causal inference, calibrating GBD models, and tailoring interventions to the Jordanian population's evolving needs.

## 5 Conclusion(s)

The findings provide evidence for significant shifts in the country's health profile, with substantial declines in age‐standardized health loss and premature mortality alongside the increasing prominence of NCDs. Ischemic heart disease and diabetes mellitus were the two leading Level 3 causes of DALYs in 2023, while metabolic risks remained major contributors to the national disease burden. Although males continued to experience higher DALY rates than females, the difference between the sexes narrowed over time. These changes exemplify the need for focused public health strategies, including primordial and primary prevention of NCDs across the life course, continued surveillance, and equitable access to long‐term management of chronic conditions.

## Author Contributions

Yazan A. Al‐Ajlouni and Basile Njei conceived the study. Yazan A. Al‐Ajlouni and Abdulrahman Al‐Shamari curated and analyzed the GBD data, accessed and verified the underlying data, and contributed to methodology and visualization. Omar Al Ta'ani and Sara Al Zubi also accessed and verified the data. Yazan A. Al‐Ajlouni, Omar Al Ta'ani, Sara Al Zubi, and Basile Njei drafted the initial version of the manuscript. Jude Alawa, Aditi Hazra, Kaveh Khoshnood, Omar Shamieh, Nour Makarem, and Adam Coutts contributed to data interpretation and writing – review and editing. Basile Njei supervised the study and provided project administration support. All authors reviewed and approved the final manuscript and agree to be accountable for all aspects of the work.

## Funding

The authors have nothing to report.

## Conflicts of Interest

The authors declare no conflicts of interest.

## Transparency Statement

The corresponding author, Basile Njei, affirms that this manuscript is an honest, accurate, and transparent account of the study being reported; that no important aspects of the study have been omitted; and that any discrepancies from the study as planned have been explained.

## Supporting information


**Figure S1A:** Annual distribution of the three broad GBD cause categories in the all‐age prevalence burden, Jordan, 1990–2023. Note. The plotted shares describe the distribution of the summed Level 1 category estimates. Prevalence categories can overlap at the person level and should not be interpreted as mutually exclusive proportions of individuals.
**Figure S1B:** Annual distribution of deaths across CMNN conditions, non‐communicable diseases, and injuries in Jordan, 1990–2023. *Note:* Shares were calculated from all‐age numbers for both sexes.
**Figure S1C:** Annual distribution of DALYs across CMNN conditions, non‐communicable diseases, and injuries in Jordan, 1990–2023. *Note:* Shares were calculated from all‐age numbers for both sexes.
**Figure S2A:** All‐age prevalence rates for the three broad GBD cause categories in Jordan, 2023. *Note:* Rates are per 100,000 for both sexes. Category‐specific prevalence estimates can overlap at the person level.
**Figure S2B:** All‐age death rates for the three broad GBD cause categories in Jordan, 2023. *Note:* Rates are per 100,000 for both sexes.
**Figure S2C:** All‐age DALY rates for the three broad GBD cause categories in Jordan, 2023. *Note:* Rates are per 100,000 for both sexes.
**Figure S3A:** Age composition of the 20 leading Level 3 causes of prevalent cases in Jordan, 2023. *Note:* Stacked values are numbers, not sums of age‐specific rates. Age bands are non‐overlapping. Cause‐specific prevalence should not be summed to estimate the number of unique people with any condition.
**Figure S3B:** Age composition of the 20 leading Level 3 causes of death in Jordan, 2023. *Note:* Stacked values are numbers. All age bands are non‐overlapping.
**Figure S3C:** Age composition of the 20 leading Level 3 causes of DALYs in Jordan, 2023. *Note:* Stacked values are numbers. All age bands are non‐overlapping

Supporting File 1

## Data Availability

The data is available upon reasonable request from the corresponding author. The annotated analytical code, source file inventory, filters, and reproducibility workflow used to generate the revised tables and figures are provided with the revision materials.

## References

[1] M. Ababsa , “Jordan National Urban Policy,” 2019.

[2] O. Altobaishat , M. Abouzid , D. Omari , et al., “Examining the Burden of Mental Disorders in Jordan: An Ecological Study Over Three Decades,” BMC Psychiatry 25, no. 1 (2025): 218. 10.1186/s12888-025-06658-x PMC11895291 40069668

[3] G. Salameh , D. Marais , and R. Khoury , “Impact of COVID‐19 Pandemic on Mental Health Among the Population in Jordan,” International Journal of Environmental Research and Public Health 20, no. 14 (2023): 6382. 10.3390/ijerph20146382 PMC10379603 37510614

[4] J. A. Alnsour , “Managing Urban Growth in the City of Amman, Jordan,” Cities 50 (2016): 93–99.

[5] M. M. Albattah , “Geoinformatics Engineering and GIS for Urban Growth Patterns Assessment,” Wasit Journal of Engineering Sciences 10, no. 4 (2023): 46–57.

[6] J. Al‐Bakri , S. Shawash , A. Ghanim , and R. Abdelkhaleq , “Geospatial Techniques for Improved Water Management in Jordan,” Water 8, no. 4 (2016): 132.

[7] Z. A. O. Aldeek , M. M. Alibrahim , and S. Zeadeh , “Requalifying the Irbid Refugee Camp: Towards Integrated Urban Development in Jordan,” International Journal of Sustainable Development and Planning 18, no. 9 (2023): 2769–2777.

[8] H. S. Al‐Srehan , “The Impact of Social Adjustment Policy on Syrian Refugees,” Journal of International Studies 13, no. 3 (2020): 85–97.

[9] S. Saleh , S. Ibrahim , J. L. Diab , and M. Osman , “Integrating Refugees Into National Health Systems Amid Political and Economic Constraints in the EMR: Approaches From Lebanon and Jordan,” Journal of Global Health 12 (2022): 03008. 10.7189/jogh.12.03008 PMC8932459 35356647

[10] S. Doocy , E. Lyles , L. Akhu‐Zaheya , A. Oweis , N. Al Ward , and A. Burton , “Health Service Utilization Among Syrian Refugees With Chronic Health Conditions in Jordan,” PLoS One 11, no. 4 (2016): e0150088. 10.1371/journal.pone.0150088 PMC4830531 27073930

[11] D. Al‐Hourani , M. Al‐Wriekat , R. Llamas‐Ramos , and I. Llamas‐Ramos , “The Psychological Impact Among Syrian Refugees in Host Countries,” Healthcare 13, no. 5 (2025): 488. 10.3390/healthcare13050488 PMC11899006 40077050

[12] H. N. Madanat , K. P. Troutman , and B. Al‐Madi , “The Nutrition Transition in Jordan: The Political, Economic and Food Consumption Contexts,” Promotion & Education 15, no. 1 (2008): 6–10. 10.1177/1025382307088092 18430689

[13] H. Abdel‐Razeq , F. Attiga , and A. Mansour , “Cancer Care in Jordan,” Hematology/Oncology and Stem Cell Therapy 8, no. 2 (2015): 64–70. 10.1016/j.hemonc.2015.02.001 25732671

[14] H. Alduraidi , A. Abdulla Aqel , Z. Saleh , I. Almansour , and M. Darawad , “UNRWA's Role in Promoting Health Outcomes of Palestinian Refugees in Jordan: A Systematic Literature Review,” Public Health Nursing 38, no. 4 (2021): 692–700. 10.1111/phn.12889 33715186

[15] S. Tyrovolas , C. El Bcheraoui , S. A. Alghnam , et al., “The Burden of Disease in Saudi Arabia 1990–2017: Results From the Global Burden of Disease Study 2017,” Lancet Planetary Health 4, no. 5 (2020): e195–e208. 10.1016/S2542-5196(20)30075-9 PMC7653403 32442495

[16] A. Hafeez , W. J. Dangel , S. M. Ostroff , et al., “The State of Health in Pakistan and Its Provinces and Territories, 1990–2019: A Systematic Analysis for the Global Burden of Disease Study 2019,” Lancet Global Health 11, no. 2 (2023): e229–e243. 10.1016/S2214-109X(22)00497-1 PMC10009760 36669807

[17] Y. A. Al‐Ajlouni , O. Al Ta'ani , G. Shamaileh , et al., “The Burden of Cardiovascular Diseases in Jordan: A Longitudinal Analysis From the Global Burden of Disease Study, 1990–2019,” BMC Public Health 24, no. 1 (2024): 879. 10.1186/s12889-024-18316-0 PMC10958901 38515115

[18] S. M. Gabbay , “Balancing Acts: Jordan's Remarkable Stability Amidst Regional Conflict and Humanitarian Response,” Advances in Social Sciences Research Journal 10, no. 11 (2023): 193–201.

[19] Global Burden of Disease Collaborative Network , “Global Burden of Disease Study 2023 (GBD 2023).” In: Seattle USIfHMaEI, editor, 2025.

[20] A. H. Mokdad , S. Jaber , M. I. A. Aziz , et al., “The State of Health in the Arab World, 1990–2010: An Analysis of the Burden of Diseases, Injuries, and Risk Factors,” Lancet 383, no. 9914 (2014): 309–320. 10.1016/S0140-6736(13)62189-3 24452042

[21] A. Tamimi , M. Al‐Abbadi , I. Tamimi , M. Juweid , M. Ahmad , and F. Tamimi , “The Transformation of Jordan's Healthcare System in an Area of Conflict,” BMC Health Services Research 24, no. 1 (2024): 1033. 10.1186/s12913-024-11467-1 PMC11378627 39243074

[22] Y. S. Khader , M. Alyahya , N. Al‐Sheyab , K. Shattnawi , H. R. Saqer , and A. Batieha , “Evaluation of Maternal and Newborn Health Services in Jordan,” Journal of Multidisciplinary Healthcare 11 (2018): 439–456. 10.2147/JMDH.S171982 PMC6130273 30233201

[23] B. Ala'a , S. A. Altarawneh , L. Z. A. Ghazaleh , et al., “Navigating Road Traffic Accidents in Jordan: A Retrospective Exploration of the Health and Economic Impacts,” Cureus 16, no. 5 (2024): e60497. 10.7759/cureus.60497 PMC11180447 38882988

[24] S. St Laurent , N. Khasawneh , E. Al‐Nabulsi , et al., “Development of a Country‐Wide Trauma System in Jordan,” Journal of the American College of Surgeons 231, no. 4 (2020): S122.

[25] R. Rihani , A. Awidi , M. Barbar , et al., “Advancing Paediatric Cancer Care in Jordan: A Strategic 10‐Year Roadmap,” Lancet Child & Adolescent Health 9, no. 7 (2025): 497–507. 10.1016/S2352-4642(25)00103-8 40506198

[26] K. Ajlouni , Y. Khader , A. Batieha , H. Jaddou , and M. El‐Khateeb , “An Alarmingly High and Increasing Prevalence of Obesity in Jordan,” Epidemiology and Health 42 (2020): e2020040. 10.4178/epih.e2020040 PMC7871146 32512659

[27] A. R. Al‐Nuaim , “Prevalence of Glucose Intolerance in Urban and Rural Communities in Saudi Arabia,” Diabetic Medicine 14, no. 7 (1997): 595–602. 10.1002/(SICI)1096-9136(199707)14:7<595::AID-DIA377>3.0.CO;2-C 9223399

[28] L. K. A. Subhi , S. Bose , and M. F. A. Ani , “Prevalence of Physically Active and Sedentary Adolescents in 10 Eastern Mediterranean Countries and Its Relation With Age, Sex, and Body Mass Index,” Journal of Physical Activity and Health 12, no. 2 (2015): 257–265. 10.1123/jpah.2013-0134 24763105

[29] N. T. Eley , E. Namey , K. McKenna , A. C. Johnson , and G. Guest , “Beyond the Individual: Social and Cultural Influences on the Health‐Seeking Behaviors of African American Men,” American Journal of Men's Health 13, no. 1 (2019): 1557988319829953. 10.1177/1557988319829953 PMC6440067 30767594

[30] J. Mak , S. Mayhew , Av Maercker , I. R. Team , and M. Colombini , “Men's Use of Sexual Health and HIV Services in Swaziland: A Mixed Methods Study,” Sexual Health 13, no. 3 (2016): 265–274. 10.1071/SH15244 27028455

[31] R. L. Kaplan , S. Nehme , F. Aunon , D. de Vries , and G. Wagner , “Suicide Risk Factors Among Trans Feminine Individuals in Lebanon,” International Journal of Transgenderism 17, no. 1 (2016): 23–30. 10.1080/15532739.2015.1117406 PMC5012530 27616943

[32] D. M. Griffith , K. R. Ellis , and J. Ober Allen , “How Does Health Information Influence African American Men's Health Behavior?,” American Journal of Men's Health 6, no. 2 (2011): 156–163. 10.1177/1557988311426910 22178902

[33] C. Akik , H. Ghattas , S. Mesmar , M. Rabkin , W. El‐Sadr , and F. M. Fouad , “Host Country Responses to Non‐Communicable Diseases Amongst Syrian Refugees: A Review,” Conflict and Health 13, no. 1 (2019): 8. 10.1186/s13031-019-0192-2 PMC6431037 30949232

[34] S. Doocy , E. Lyles , L. Akhu‐Zaheya , A. Burton , and G. Burnham , “Health Service Access and Utilization Among Syrian Refugees in Jordan,” International Journal for Equity in Health 15, no. 1 (2016): 108. 10.1186/s12939-016-0399-4 PMC4946096 27418336

[35] D. E. Rosenkrantz , W. W. Black , R. L. Abreu , M. E. Aleshire , and K. Fallin‐Bennett , “Health and Health Care of Rural Sexual and Gender Minorities: A Systematic Review,” Stigma and Health 2, no. 3 (2017): 229–243.

[36] J. K. Ganle , B. Obeng , A. Y. Segbefia , V. Mwinyuri , J. Y. Yeboah , and L. Baatiema , “How Intra‐Familial Decision‐Making Affects Women's Access to, and Use of Maternal Healthcare Services in Ghana: A Qualitative Study,” BMC Pregnancy and Childbirth 15, no. 1 (2015): 173. 10.1186/s12884-015-0590-4 PMC4537557 26276165

[37] Y. Alem , S. Hassen , and G. Köhlin , “Decision‐Making Within the Household: The Role of Division of Labor and Differences in Preferences,” Journal of Economic Behavior & Organization 207 (2023): 511–528.

[38] M. Daye , S. A. Temiz , Y. Durduran , et al., “Analysis of Consultation Cases Referred From Pediatrics Department to Dermatology Outpatient Clinic: Retrospective Study,” Clinical and Experimental Health Sciences 9, no. 4 (2019): 300–303.

[39] A. Musaiger , M. Al‐Mannai , and R. Tayyem , “Prevalence of Overweight and Obesity Among Female Adolescents in Jordan: A Comparison Between Two International Reference Standards,” Pakistan Journal of Medical Sciences 29, no. 2 (2013): 678–679. 10.12669/pjms.292.3222 PMC3809221 24353605

[40] M. Bustami , K. Z. Matalka , E. Mallah , et al., “The Prevalence of Overweight and Obesity Among Women in Jordan: A Risk Factor for Developing Chronic Diseases,” Journal of Multidisciplinary Healthcare 14 (2021): 1533–1541. 10.2147/JMDH.S313172 PMC8235929 34188480

[41] W. S. Alsaud , M. Tabbaa , V. Kasabri , et al., “Prevalence of Cardiovascular Diseases Risk Factors Among Jordanians,” Journal of the Saudi Heart Association 32, no. 2 (2020): 324–333. 10.37616/2212-5043.1074 PMC7640553 33154938

[42] F. I. Hawari , R. K. Bader , H. M. Beano , et al., “Perceptions of Young Jordanian Adults to Proposed Anti‐Tobacco Pictorial Warning Labels,” BMC Public Health 11, no. 1 (2011): 414. 10.1186/1471-2458-11-414 PMC3141444 21627794

[43] R. Nakkash , Y. Khader , A. Chalak , et al., “Prevalence of Cigarette and Waterpipe Tobacco Smoking Among Adults in Three Eastern Mediterranean Countries: A Cross‐Sectional Household Survey,” BMJ Open 12, no. 3 (2022): e055201. 10.1136/bmjopen-2021-055201 PMC8900030 35246419

[44] M. Jaghbir , S. Shareif , and M. Ahram , “Quitting Smoking and Utilization of Smoking Cessation Services in Jordan: A Population‐Based Survey,” Eastern Mediterranean Health Journal 20, no. 09 (2014): 538–546. 25343466

[45] S. R. Obeidat , Z. A. Malkawi , O. F. Khabour , and A. G. Alsa'di , “Prevalence, Knowledge, Attitudes, and Perceptions About E‐Cigarette Smoking Among Students in the Dental Fields in Jordan,” International Journal of Dentistry 2025, no. 1 (2025): 6521183. 10.1155/ijod/6521183 PMC11961272 40170985

