# Supplementary material for: The State of Health in Jordan, 1990–2023: A Systematic Analysis for the Global Burden of Disease Study
Source: Health Sci Rep. 2026 Oct 31;9(11):e73461. doi: 10.1002/hsr2.73461 (PMC13723306; doi:10.1002/hsr2.73461)
Supplement: Supplementary file 1 [file HSR2-9-e73461-s001.docx]

**Figure 1**

**Figure 1.** Trends in age-standardized DALY, YLL, and YLD rates in Jordan by sex, 1990-2023.

Note. Rates are per 100,000 population. DALYs equal YLLs plus YLDs. The corrected panels use the source measure corresponding to each y-axis.

**Figure 2**

**Figure 2.** Top 14 mutually exclusive Level 3 causes of DALYs in Jordan in 1990 and 2023, with changes in all-age and age-standardized rates.

Note. Rankings use all-age DALY rates for both sexes. Solid connecting lines indicate an increase and dashed lines indicate a decrease in the all-age DALY rate. Percentage changes include 95% uncertainty intervals.

**Figure 3**

**Figure 3.** Leading Level 3 causes of DALYs and deaths in Jordan by non-overlapping age group and sex, 2023.

Note. Age-specific panels display the four leading causes in each grouped age band. Sex-specific panels display the ten leading age-standardized causes for both sexes, females, and males. Blank cells mean that a cause was not within the displayed top causes, not that data were missing.

**Figure 4A**

**Figure 4A.** Comparison of age-standardized DALY rates for Jordan's 20 leading Level 3 DALY causes with regional and global estimates, 2023.

Note. Causes were selected independently using Jordan's DALY ranking. Comparator values are GBD aggregate estimates, not simple arithmetic averages.

**Figure 4B**

**Figure 4B.** Comparison of age-standardized death rates for Jordan's 20 leading Level 3 causes of death with regional and global estimates, 2023.

Note. Causes were selected independently using Jordan's death ranking; consequently, the cause set differs from panel A. Comparator values are GBD aggregate estimates.

**Figure 5A**

**Figure 5A.** Leading leaf risk factors contributing to age-standardized DALY rates in Jordan, 2023, decomposed by Level 2 cause group.

Note. Risk factors are terminal nodes in the GBD risk hierarchy and were selected independently for DALYs. Attributable burdens overlap across risk factors and must not be summed across rows.

**Figure 5B**

**Figure 5B.** Leading leaf risk factors contributing to age-standardized death rates in Jordan, 2023, decomposed by Level 2 cause group.

Note. Risk factors were selected independently for deaths. Attributable burdens overlap across risk factors and must not be summed across rows.

**Figure S1A**

**Figure S1A.** Annual distribution of the three broad GBD cause categories in the all-age prevalence burden, Jordan, 1990-2023.

Note. The plotted shares describe the distribution of the summed Level 1 category estimates. Prevalence categories can overlap at the person level and should not be interpreted as mutually exclusive proportions of individuals.

**Figure S1B**

**Figure S1B.** Annual distribution of deaths across CMNN conditions, non-communicable diseases, and injuries in Jordan, 1990-2023.

Note. Shares were calculated from all-age numbers for both sexes.

**Figure S1C**

**Figure S1C.** Annual distribution of DALYs across CMNN conditions, non-communicable diseases, and injuries in Jordan, 1990-2023.

Note. Shares were calculated from all-age numbers for both sexes.

**Figure S2A**

**Figure S2A.** All-age prevalence rates for the three broad GBD cause categories in Jordan, 2023.

Note. Rates are per 100,000 for both sexes. Category-specific prevalence estimates can overlap at the person level.

**Figure S2B**

**Figure S2B.** All-age death rates for the three broad GBD cause categories in Jordan, 2023.

Note. Rates are per 100,000 for both sexes.

**Figure S2C**

**Figure S2C.** All-age DALY rates for the three broad GBD cause categories in Jordan, 2023.

Note. Rates are per 100,000 for both sexes.

**Figure S3A**

**Figure S3A.** Age composition of the 20 leading Level 3 causes of prevalent cases in Jordan, 2023.

Note. Stacked values are numbers, not sums of age-specific rates. Age bands are non-overlapping. Cause-specific prevalence should not be summed to estimate the number of unique people with any condition.

**Figure S3B**

**Figure S3B.** Age composition of the 20 leading Level 3 causes of death in Jordan, 2023.

Note. Stacked values are numbers. All age bands are non-overlapping.

**Figure S3C**

**Figure S3C.** Age composition of the 20 leading Level 3 causes of DALYs in Jordan, 2023.

Note. Stacked values are numbers. All age bands are non-overlapping.
