# Supplementary material for: The State of Health in Jordan, 1990–2023: A Systematic Analysis for the Global Burden of Disease Study
Source: Health Sci Rep. 2026 Oct 31;9(11):e73461. doi: 10.1002/hsr2.73461 (PMC13723306; doi:10.1002/hsr2.73461)
Supplement: Supplementary file 2 [file HSR2-9-e73461-s002.docx]

**Supplementary Table S1. Definitions and hierarchical classification of causes, risk factors, and measures included in the analysis**

Cause rankings were restricted to mutually exclusive GBD Level 3 categories. Part A lists the Level 3 causes shown in Table 2 and Figures 2–4 and S3. Part B lists selected Level 4 causes and their parent categories. Parts C and D define the risk factors, measures, and analytical terms used in the tables and figures.

**Part A. Level 3 causes used in the tables and figures**

| **Cause** | **GBD level** | **Immediate parent category** | **Included Level 4 subcauses** | Exclusions | GBD cause ID |
| --- | --- | --- | --- | --- | --- |
| Sexually transmitted infections excluding HIV | Level 3 | HIV/AIDS and sexually transmitted infections | Syphilis; Chlamydial infection; Gonococcal infection; Trichomoniasis; Genital herpes; Other sexually transmitted infections | None | 393 |
| Lower respiratory infections | Level 3 | Respiratory infections and tuberculosis | No Level 4 subcause is listed under this category in the supplied hierarchy. | None | 322 |
| COVID-19 | Level 3 | Respiratory infections and tuberculosis | No Level 4 subcause is listed under this category in the supplied hierarchy. | None | 1048 |
| Diarrheal diseases | Level 3 | Enteric infections | No Level 4 subcause is listed under this category in the supplied hierarchy. | None | 302 |
| Pertussis | Level 3 | Other infectious diseases | No Level 4 subcause is listed under this category in the supplied hierarchy. | None | 339 |
| Neonatal disorders | Level 3 | Maternal and neonatal disorders | Neonatal preterm birth; Neonatal encephalopathy due to birth asphyxia and trauma; Neonatal sepsis and other neonatal infections; Hemolytic disease and other neonatal jaundice; Other neonatal disorders | None | 380 |
| Dietary iron deficiency | Level 3 | Nutritional deficiencies | No Level 4 subcause is listed under this category in the supplied hierarchy. | None | 390 |
| Stomach cancer | Level 3 | Neoplasms | No Level 4 subcause is listed under this category in the supplied hierarchy. | None | 414 |
| Colon and rectum cancer | Level 3 | Neoplasms | No Level 4 subcause is listed under this category in the supplied hierarchy. | None | 441 |
| Pancreatic cancer | Level 3 | Neoplasms | No Level 4 subcause is listed under this category in the supplied hierarchy. | None | 456 |
| Tracheal, bronchus, and lung cancer | Level 3 | Neoplasms | No Level 4 subcause is listed under this category in the supplied hierarchy. | None | 426 |
| Breast cancer | Level 3 | Neoplasms | No Level 4 subcause is listed under this category in the supplied hierarchy. | None | 429 |
| Prostate cancer | Level 3 | Neoplasms | No Level 4 subcause is listed under this category in the supplied hierarchy. | None | 438 |
| Leukemia | Level 3 | Neoplasms | Acute lymphoid leukemia; Chronic lymphoid leukemia; Acute myeloid leukemia; Chronic myeloid leukemia; Other leukemia | None | 487 |
| Ischemic heart disease | Level 3 | Cardiovascular diseases | No Level 4 subcause is listed under this category in the supplied hierarchy. | None | 493 |
| Stroke | Level 3 | Cardiovascular diseases | Ischemic stroke; Intracerebral hemorrhage; Subarachnoid hemorrhage | None | 494 |
| Hypertensive heart disease | Level 3 | Cardiovascular diseases | No Level 4 subcause is listed under this category in the supplied hierarchy. | None | 498 |
| Chronic obstructive pulmonary disease | Level 3 | Chronic respiratory diseases | No Level 4 subcause is listed under this category in the supplied hierarchy. | None | 509 |
| Cirrhosis and other chronic liver diseases | Level 3 | Digestive diseases | Chronic hepatitis B including cirrhosis; Chronic hepatitis C including cirrhosis; Cirrhosis due to alcohol; Nonalcoholic fatty liver disease including cirrhosis; Cirrhosis due to other causes | None | 521 |
| Upper digestive system diseases | Level 3 | Digestive diseases | Peptic ulcer disease; Gastritis and duodenitis; Gastroesophageal reflux disease | None | 992 |
| Alzheimer's disease and other dementias | Level 3 | Neurological disorders | No Level 4 subcause is listed under this category in the supplied hierarchy. | None | 543 |
| Headache disorders | Level 3 | Neurological disorders | Migraine; Tension-type headache | None | 972 |
| Depressive disorders | Level 3 | Mental disorders | Major depressive disorder; Dysthymia | None | 567 |
| Anxiety disorders | Level 3 | Mental disorders | No Level 4 subcause is listed under this category in the supplied hierarchy. | None | 571 |
| Diabetes mellitus | Level 3 | Diabetes and kidney diseases | Diabetes mellitus type 1; Diabetes mellitus type 2 | None | 587 |
| Chronic kidney disease | Level 3 | Diabetes and kidney diseases | Chronic kidney disease due to diabetes mellitus type 1; Chronic kidney disease due to diabetes mellitus type 2; Chronic kidney disease due to hypertension; Chronic kidney disease due to glomerulonephritis; Chronic kidney disease due to other and unspecified causes | None | 589 |
| Dermatitis | Level 3 | Skin and subcutaneous diseases | Atopic dermatitis; Contact dermatitis; Seborrhoeic dermatitis | None | 654 |
| Age-related and other hearing loss | Level 3 | Sense organ diseases | No Level 4 subcause is listed under this category in the supplied hierarchy. | None | 674 |
| Osteoarthritis | Level 3 | Musculoskeletal disorders | Osteoarthritis hip; Osteoarthritis knee; Osteoarthritis hand; Osteoarthritis other | None | 628 |
| Low back pain | Level 3 | Musculoskeletal disorders | No Level 4 subcause is listed under this category in the supplied hierarchy. | None | 630 |
| Neck pain | Level 3 | Musculoskeletal disorders | No Level 4 subcause is listed under this category in the supplied hierarchy. | None | 631 |
| Other musculoskeletal disorders | Level 3 | Musculoskeletal disorders | No Level 4 subcause is listed under this category in the supplied hierarchy. | None | 639 |
| Congenital birth defects | Level 3 | Other non-communicable diseases | Neural tube defects; Congenital heart anomalies; Orofacial clefts; Down syndrome; Turner syndrome; Klinefelter syndrome; Other chromosomal abnormalities; Congenital musculoskeletal and limb anomalies; Urogenital congenital anomalies; Digestive congenital anomalies; Other congenital birth defects | None | 641 |
| Gynecological diseases | Level 3 | Other non-communicable diseases | Uterine fibroids; Polycystic ovarian syndrome; Female infertility; Endometriosis; Genital prolapse; Premenstrual syndrome; Other gynecological diseases | None | 603 |
| Hemoglobinopathies and hemolytic anemias | Level 3 | Other non-communicable diseases | Thalassemias; Thalassemias trait; Sickle cell disorders; Sickle cell trait; G6PD deficiency; G6PD trait; Other hemoglobinopathies and hemolytic anemias | None | 613 |
| Endocrine, metabolic, blood, and immune disorders | Level 3 | Other non-communicable diseases | Thyroid diseases; Other endocrine, metabolic, blood, and immune disorders | None | 619 |
| Oral disorders | Level 3 | Other non-communicable diseases | Caries of deciduous teeth; Caries of permanent teeth; Periodontal diseases; Edentulism; Other oral disorders | None | 680 |
| Road injuries | Level 3 | Transport injuries | Pedestrian road injuries; Cyclist road injuries; Motorcyclist road injuries; Motor vehicle road injuries; Other road injuries | None | 689 |
| Falls | Level 3 | Unintentional injuries | No Level 4 subcause is listed under this category in the supplied hierarchy. | None | 697 |
| Drowning | Level 3 | Unintentional injuries | No Level 4 subcause is listed under this category in the supplied hierarchy. | None | 698 |
| Other unintentional injuries | Level 3 | Unintentional injuries | No Level 4 subcause is listed under this category in the supplied hierarchy. | None | 716 |
| Interpersonal violence | Level 3 | Self-harm and interpersonal violence | Physical violence by firearm; Physical violence by sharp object; Sexual violence; Physical violence by other means | None | 724 |

**Part B. Selected Level 4 causes and their parent categories**

| **Official GBD term** | **GBD level** | **Immediate parent category** | **Definition and relationship to parent** | Exclusions | GBD cause ID |
| --- | --- | --- | --- | --- | --- |
| Neonatal preterm birth | Level 4 | Neonatal disorders | A Level 4 subcause within Neonatal disorders. It is included when the Level 3 parent is reported and is not ranked beside that parent. | None | 381 |
| Intracerebral hemorrhage | Level 4 | Stroke | A Level 4 subcause within Stroke. It is included when the Level 3 parent is reported and is not ranked beside that parent. | None | 496 |
| Diabetes mellitus type 2 | Level 4 | Diabetes mellitus | A Level 4 subcause within Diabetes mellitus. It is included when the Level 3 parent is reported and is not ranked beside that parent. | None | 976 |
| Congenital heart anomalies | Level 4 | Congenital birth defects | A Level 4 subcause within Congenital birth defects. It is included when the Level 3 parent is reported and is not ranked beside that parent. | None | 643 |
| Other congenital birth defects | Level 4 | Congenital birth defects | A Level 4 subcause within Congenital birth defects. It is included when the Level 3 parent is reported and is not ranked beside that parent. | None | 652 |
| Pedestrian road injuries | Level 4 | Road injuries | A Level 4 subcause within Road injuries. It is included when the Level 3 parent is reported and is not ranked beside that parent. | None | 690 |
| Motor vehicle road injuries | Level 4 | Road injuries | A Level 4 subcause within Road injuries. It is included when the Level 3 parent is reported and is not ranked beside that parent. | None | 693 |

**Part C. Risk factors used in Table 3 and Figure 5**

Figure 5 includes terminal (leaf) risk factors to avoid ranking parent and child risks together. Estimates for different risks may overlap because the same health outcome can be attributable to more than one exposure.

| **Risk factor** | **GBD level** | **Immediate parent category** | **Definition used in this analysis** | **Where shown** | **REI ID** |
| --- | --- | --- | --- | --- | --- |
| Ambient particulate matter pollution | Level 4 | Particulate matter pollution | Burden attributable to exposure to outdoor particulate matter above the GBD minimum-risk exposure distribution. | Table 3; Figure 5A; Figure 5B | 86 |
| Low temperature | Level 3 | Non-optimal temperature | Burden attributable to temperatures below the GBD minimum-risk exposure distribution for the location and period. | Table 3; Figure 5A; Figure 5B | 338 |
| Lead exposure | Level 3 | Other environmental risks | Burden attributable to exposure to lead above the GBD minimum-risk exposure distribution. | Figure 5A; Figure 5B | 91 |
| Short gestation | Level 4 | Low birth weight and short gestation | Burden attributable to gestational age below the GBD minimum-risk exposure distribution. | Table 3; Figure 5A | 334 |
| Low birth weight | Level 4 | Low birth weight and short gestation | Burden attributable to birth weight below the GBD minimum-risk exposure distribution. | Table 3; Figure 5A; Figure 5B | 335 |
| Iron deficiency | Level 3 | Child and maternal malnutrition | Burden attributable to inadequate iron exposure as defined in the GBD comparative risk assessment. | Table 3; Figure 5A | 95 |
| Smoking | Level 3 | Tobacco | Burden attributable to current or former use of smoked tobacco products, as defined in the GBD risk framework. | Table 3; Figure 5A; Figure 5B | 99 |
| Secondhand smoke | Level 3 | Tobacco | Burden attributable to exposure to tobacco smoke produced by other people. | Table 3; Figure 5A; Figure 5B | 100 |
| Diet low in fruits | Level 3 | Dietary risks | Burden attributable to fruit intake below the GBD minimum-risk exposure distribution. | Table 3; Figure 5A; Figure 5B | 111 |
| Diet low in vegetables | Level 3 | Dietary risks | Burden attributable to vegetable intake below the GBD minimum-risk exposure distribution. | Table 3; Figure 5A; Figure 5B | 112 |
| Diet low in whole grains | Level 3 | Dietary risks | Burden attributable to whole-grain intake below the GBD minimum-risk exposure distribution. | Table 3; Figure 5A; Figure 5B | 113 |
| Diet low in legumes | Level 3 | Dietary risks | Burden attributable to legume intake below the GBD minimum-risk exposure distribution. | Figure 5B | 333 |
| Diet low in nuts and seeds | Level 3 | Dietary risks | Burden attributable to intake of nuts and seeds below the GBD minimum-risk exposure distribution. | Figure 5B | 114 |
| Diet low in fiber | Level 3 | Dietary risks | Burden attributable to fiber intake below the GBD minimum-risk exposure distribution. | Figure 5B | 119 |
| Diet low in seafood omega-3 fatty acids | Level 3 | Dietary risks | Burden attributable to intake of seafood omega-3 fatty acids below the GBD minimum-risk exposure distribution. | Figure 5A; Figure 5B | 121 |
| Diet high in sodium | Level 3 | Dietary risks | Burden attributable to sodium intake above the GBD minimum-risk exposure distribution. | Figures 5A and 5B | 124 |
| Sexual violence against children | Level 3 | Sexual violence against children and bullying | Burden attributable to exposure to sexual violence during childhood, as defined in the GBD risk framework. | Figure 5A | 134 |
| Low physical activity | Level 2 | Behavioral risks | Burden attributable to physical activity below the GBD minimum-risk exposure distribution. | Figure 5A; Figure 5B | 125 |
| High fasting plasma glucose | Level 2 | Metabolic risks | Burden attributable to fasting plasma glucose above the GBD minimum-risk exposure distribution. | Table 3; Figure 5A; Figure 5B | 105 |
| High LDL cholesterol | Level 2 | Metabolic risks | Burden attributable to LDL cholesterol above the GBD minimum-risk exposure distribution. | Table 3; Figure 5A; Figure 5B | 367 |
| High systolic blood pressure | Level 2 | Metabolic risks | Burden attributable to systolic blood pressure above the GBD minimum-risk exposure distribution. | Table 3; Figure 5A; Figure 5B | 107 |
| High body-mass index | Level 2 | Metabolic risks | Burden attributable to body-mass index above the GBD minimum-risk exposure distribution. | Table 3; Figure 5A; Figure 5B | 108 |
| Kidney dysfunction | Level 2 | Metabolic risks | Burden attributable to impaired kidney function above the GBD minimum-risk exposure distribution. | Table 3; Figure 5A; Figure 5B | 341 |

**Part D. Measures and analytical terms**

| **Measure or term** | **Definition used in this analysis** | **Where used** |
| --- | --- | --- |
| Deaths | Fatal outcomes reported as either a number or a rate for the selected cause, age, sex, location, and year. | Tables 1-3; Figures 1, 3-5, S1-S3 |
| Prevalence | The proportion of the population living with a condition at a specified time. The all-cause prevalence result is a GBD point-prevalence estimate and is not the sum of cause-specific prevalence estimates or the percentage of unique people with any condition. | Table 1; Figures 1 and S3 |
| Incidence | The occurrence of new cases during a specified period, reported as a number or rate. | Methods and source analysis |
| Years of life lost (YLLs) | Premature fatal health loss, calculated by multiplying deaths at each age by the remaining standard life expectancy at that age. | Table 1; Figure 1 |
| Years lived with disability (YLDs) | Non-fatal health loss calculated from condition prevalence and the corresponding disability weight, with adjustment for comorbidity in the GBD estimation process. | Table 1; Figure 1 |
| Disability-adjusted life years (DALYs) | Total health loss from fatal and non-fatal outcomes, calculated as YLLs plus YLDs. | Tables 1-3; Figures 1-5 and S1-S3 |
| Number | The absolute estimated count of events or health-loss units in the selected population. | Figures 5 and S3; source analyses |
| Rate per 100,000 | The estimated number of events or health-loss units divided by the relevant population and multiplied by 100,000. | Tables 1-3; Figures 1-4, S1 and S2 |
| All-age rate | A population rate calculated across all ages without age standardization. It reflects both the level of burden and the population age structure. | Tables 2 and 3 |
| Age-standardized rate | A rate standardized to the GBD world standard population to allow comparisons across years and populations with different age structures. | Tables 1-3; Figures 1-4, S1 and S2 |
| Percent change | The relative change between the starting and ending estimates, calculated as ((ending value - starting value) / starting value) x 100. | Tables 1-3; Figures 1, 2, S1 and S2 |
| Rank | The position obtained after ordering estimates from highest to lowest within the specified measure, year, age, sex, and geographic stratum. Cause rankings use Level 3 causes only; Figure 5 uses leaf risk factors only. | Table 2; Figures 2-5 and S3 |
| 95% uncertainty interval | The interval bounded by the 2.5th and 97.5th percentiles of the GBD uncertainty draws. It reflects uncertainty in data inputs and model estimation. | Tables 1-3 and reported Results |
| Risk-attributable burden | The estimated reduction in deaths or DALYs that would occur if exposure were reduced to the GBD theoretical minimum-risk exposure distribution. Estimates for different risks may overlap and should not be added together. | Table 3; Figure 5 |
| Both sexes | The estimate for males and females combined, rather than the arithmetic mean of the two sex-specific rates. | All principal tables and figures |
| All ages | The estimate covering the complete age range included in GBD for the selected measure. | Tables 2 and 3; Figures 2 and 5 |

**Notes**

Cause names, IDs, hierarchy levels, parent categories, and parent–child relationships were taken from the IHME GBD 2023 Cause, REI, and Location Hierarchies workbook (release date October 23, 2025). Included subcauses are the immediate Level 4 children listed in that workbook. Each Level 3 estimate includes the burden assigned to its constituent subcauses, and the Level 3 categories are mutually exclusive within the rankings.

No ICD-9 or ICD-10 codes were applied directly because the analysis used published GBD estimates selected by GBD cause ID rather than individual coded records. ICD harmonisation, garbage-code redistribution, and mapping to GBD causes were undertaken by IHME.

Risk names, REI IDs, hierarchy levels, and parent categories were taken from the REI Hierarchy sheet of the same GBD 2023 release. Risk definitions follow the GBD comparative risk-assessment framework. Exposure thresholds and theoretical minimum-risk exposure distributions vary by risk, measure, age, sex, year, and location.
